# Exploring the Abnormal Modulation of the Autonomic Systems during Nasal Flow Limitation in Upper Airway Resistance Syndrome by Hilbert–Huang Transform

**DOI:** 10.3389/fmed.2017.00161

**Published:** 2017-09-28

**Authors:** Chen Lin, Men-Tzung Lo, Christian Guilleminault

**Affiliations:** ^1^Stanford University Sleep Medicine Division, Stanford University, Redwood, CA, United States; ^2^Department of Biomedical Sciences and Engineering, National Central University, Taoyuan, Taiwan

**Keywords:** parasympathetic tone, sleep, upper-airway-resistance-syndrome, Hilbert–Huang transform, daytime hypervagotony

## Abstract

Patients with nasal flow limitation and upper airway resistance syndrome (UARS) during sleep can present with low blood pressure and disturbing symptoms associated with hypervagotony. We hypothesized that the dynamic changes of the autonomic system related to inspiratory flow limitation can be quantified by the developed analytic technique applied on beat-to-beat heart rate (RR intervals) and finger photoplethysmography (PPG).

**Method:**

A breath-by-breath investigation based on the Hilbert–Huang transform was performed to explore autonomic nervous system changes observed during inspiratory flow limitation. Autonomic status was quantified from beat-to-beat heart rate analysis by high frequency (RR_HF_; 0.15–0.4 Hz), low frequency (RR_LF_; 0.04–0.15 Hz), and LF/HF ratio of each respiratory cycle. Based on respiratory-related mechanisms contained in the PPG signal, we further quantified the respiratory-related oscillations (PPG_res_). Based on esophageal pressure and nasal flow measurements, each respiratory cycle was identified and breathing patterns were classified into one of four groups: normal, inspiratory flow limitation cycles without increased effort [FL(−)], minimal inspiratory flow limitation with effort, and inspiratory flow limitation cycles with increased effort [FL(+)]. The resulting quantitative parameters of the identified cycles were calculated.

**Results:**

49 UARS patients (12 males; aged 26.8 ± 5.8 years) with apnea–hypopnea index (AHI) 3.1 ± 1.5 per hour and nine aged matched control subjects (3 males; aged 27.8 ± 4.0 years) with AHI 0.8 ± 1.1 per hour were retrospectively identified. Compared to the control group, hyperactivation of the parasympathetic system was noted during stage 2 NREM sleep by RR_HF_ (27.8 ± 18.2 vs 22.5 ± 11.12, *p* < 0.05) in 49 UARS patients. Analysis of the different classifications of respiratory cycles indicated that during “high” (increased) respiratory efforts, the RR_HF_ and PPG_res_ were significantly higher compared to “normal cycle” and “FL(−)” groups. The RR_LF_/RR_HF_ (an index of sympathetic activity) was significantly lower in the “FL(+)” group (1.66 ± 0.80) than in the “normal cycle” (1.93 ± 0.97, *p* < 0.05) and “FL(−)” groups (2.01 ± 1.01, *p* < 0.05).

**Conclusion:**

The proposed algorithm allows quantifying the temporal changes of specific mechanisms of the autonomic system on breath-by-breath basis. With no or very limited impact on oxygen saturation, the hyperactivation of parasympathetic system in associated with inspiratory flow limitation or increased respiratory efforts during stage 2 NREM sleep has been presented in this study.

## Introduction

Flow limitation is a term used when analyzing polysomnography (PSG) and monitoring nasal flow with a nasal cannula/pressure transducer system (1, 2). Most commonly, inspiratory flow limitation is the studied variable, and inspiratory flow decreases as upper airway resistance increases without clear drops of oxygen saturation and without association with the American Academy of Sleep Medicine (AASM)-defined “sleep-hypopnea”(3). Patients with such PSG-findings have clear clinical complaints particularly involving poor sleep. This clinical syndrome has been previously well described as “upper airway resistance syndrome (UARS)” (3–5). Recently, Palombini et al. performed an epidemiological investigation of the frequency of inspiratory flow limitation in a representative sample of the general Sao Paulo population and proposed criteria to define the degree of pathologic flow imitation for those patients who do not have obstructive sleep apnea by apnea and hypopnea criteria (6). The authors indicate that patients with pathological amounts of flow limitation not only complain of their sleep but already have changes to lipid and glucose metabolisms as assessed by simple blood tests (6).

In 2000, a study of more than 200 patients with complaints indicative of UARS and inspiratory flow limitation on PSG found that many subjects had low blood pressure during wakefulness and other symptoms seen with hypervagotony (3, 7) such as cold hands and feet and abnormal tilt table testing. This is in contrast to the abnormal sleep-related activation of sympathetic nervous system associated with sleep apnea–hypopnea syndrome (8–10). Such findings were confirmed in further studies (11, 12). Although the abnormal activation of the sympathetic tone and its cortege of complications during the 24 h related to repetitive drops in oxygen in association with obstructive apneas and hypopneas is well recognized, the impact of repetitive inspiratory-flow-limitation without the required minimum drops in oxygen saturation on the autonomic nervous system (ANS) is not clearly understood. In addition, the development of symptoms in UARS patients usually associated with chronic vagal nerve stimulation is not well elucidated. Two fundamental studies have suggested that a functional sympathetic de-innervation could be induced by specific manipulations of breathing during wakefulness with direct involvement of the lung inflation reflex (13, 14). However, inspiratory flow limitation during sleep involves a complex, dynamic interaction between changes in upper airway anatomy and local neuromuscular control (15). Furthermore, the nasal flow limitation events, even if they last up to several minutes, are interrupted and recur during the entire night, particularly during NREM sleep. To address the question of the interaction between flow limitation and ANS changes during NREM sleep, we developed a new analytic technique rarely used in the field of sleep and breathing and based on Hilbert–Huang transform (HHT).

We hypothesized that the dynamic changes related to inspiratory flow limitation on beat-to-beat heart rate (RR intervals) and finger photoplethysmography (PPG) signal can be quantified by the developed analytic technique; we further hypothesized that the patients with upper airway respiratory resistance without oxygen desaturation have higher parasympathetic activity as suggested (13, 14) during NREM sleep compared to healthy controls. We derived the nasal flow curve and the concomitant recording of the esophageal pressure curve from recordings during all-night stage 2 NREM sleep. We further developed a breath-by-breath analysis of RR intervals and PPG signals looking at the differences between normal breaths and inspiratory flow limited breaths monitored in patients diagnosed with UARS.

## Materials and Methods

The analyses were performed on nocturnal recordings from two different groups of subjects: patients and normal controls. We have standardized set of questions about “patient complaints” that all MDs ask at time of clinical evaluation when a patient is seen for “sleep-disorders” and same questions are asked to subjects considered as potential controls. The controls did not present sleep-related complaints at the standardized interview and further confirmed with Epworth Sleepiness Scale (ESS; 9 ± 1.5 for patients and 3 ± 2 for controls, *p* < 0.05).

### Patients

All subjects were seen successively at the Stanford Sleep Disorders Center during a 4-month period for complaints of poor sleep, tiredness, fatigue, some degree of daytime sleepiness, and other symptoms associated with sleep-related inspiratory flow limitation and “UARS.” The patients had no other clinical complaints indicating another sleep disorder and underwent a PSG confirming a normal obstructive sleep apnea–hypopnea-index (AHI) following the AASM guidelines (16), but presence of an abnormal amount of inspiratory flow limitation associated with EEG disturbances (3). To be included in the retrospective analysis, subjects must have been between 18 and 45 years of age, have a body mass index (BMI) below 30 kg/m^2^ (definition of obesity by the World Health Organization) and without other medical problems or chronic medication intake. Following a positive diagnosis, all subjects must have demonstrated clinical improvement and elimination of flow-limitation and sleep EEG disturbances following treatment with continuous positive airway pressure for 3–6 weeks.

### Control Subjects

Individuals had been recruited from the general population, without sleep complaints and with normal body habitus and normal clinical evaluation. Subjects were retrospectively selected who met the same entry criteria (age, BMI) and had PSG performed using the same recording protocol.

### Polysomnography

All subjects underwent the same PSG protocol: nocturnal PSG for a minimum of 7 h with lights out at their usual bedtime. The standard recording consisted of 4 EEG leads, 2 electrooculograms, chin and leg EMGs, and 1 ECG lead. Respiration was monitored with nasal cannula pressure-transducer, mouth thermistor, thoracic and abdominal inductive-plethysmography bands, finger oxygen saturation (Massimo™ oximeter), neck microphone, and with an esophageal pressure transducer (Pes).

All PSG data were retrieved, anonymized, and then scored blind to the patient/control status by one investigator using the AASM 2012 scoring criteria (16). These anonymized PSGs are the source of the presented analysis. This retrospective study, performed on data rendered anonymous prior to analysis, was approved by the Stanford University Medical Institutional Review Board.

### Tool: HHT As a High Temporal Resolution Analysis of a Physiological Signal

**Figure 1:**
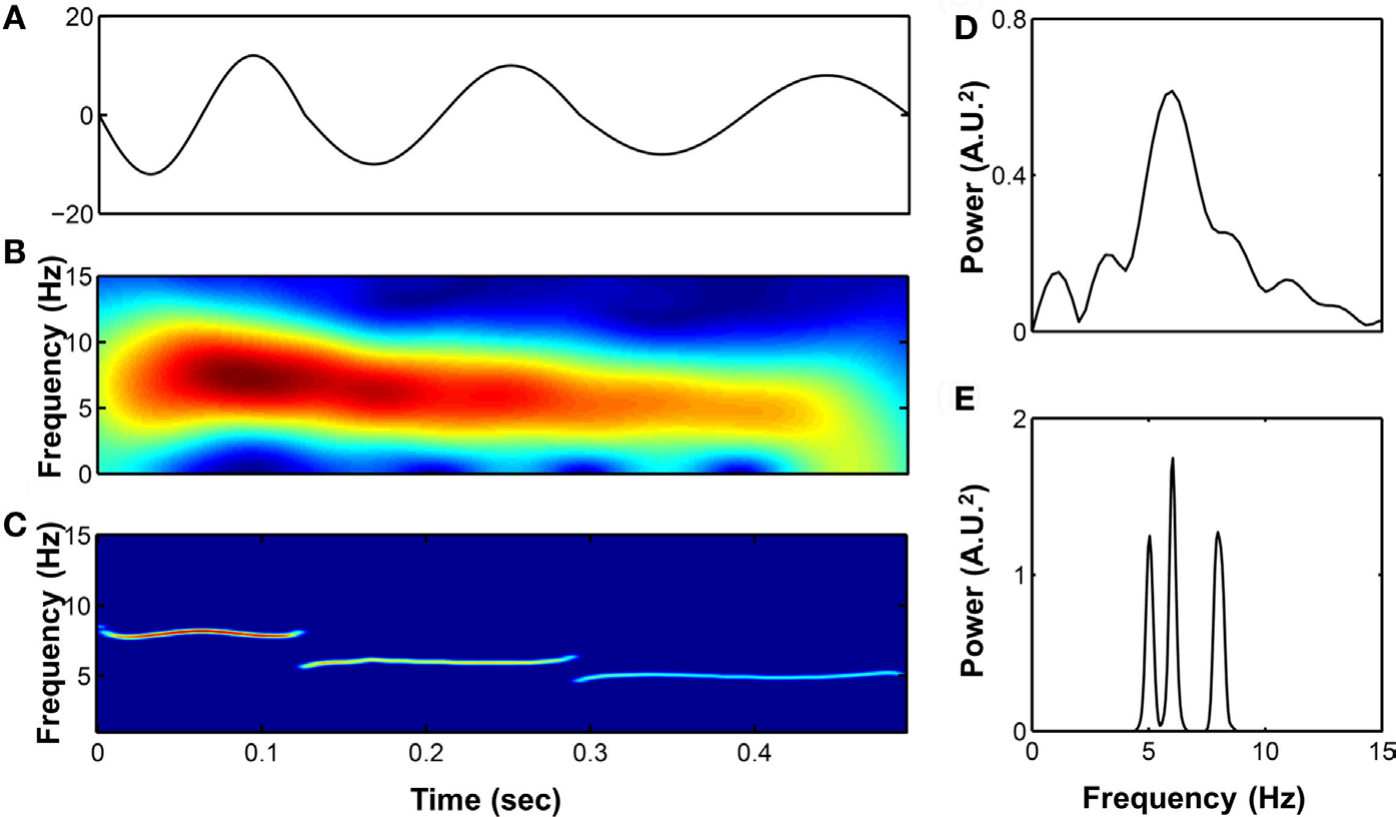
**(A)** A synthetic signal composed by three wavelets with different frequencies stitched together (8, 6, and 5 Hz, respectively). Its time–frequency representation were analyzed by short-time Fourier transform **(B)** and Hilbert–Huang transform (HHT) **(C)** as well as the power spectrum calculated by fast Fourier transform **(D)** and marginal spectrum calculated by HHT **(E)**.

The detailed algorithm of HHT has previously been well described (17). The essential component of this method is the empirical mode decomposition (EMD), which decomposes the target signals into different intrinsic mode functions (IMFs). Each IMF is symmetric with 0 mean and the instantaneous phase (frequency) and amplitudes can be further derived by the Hilbert transform. The time frequency representation of the signals can be better reconstructed compared to a Fourier-based method. For example, consider a synthetic signal with three wavelets with different frequencies (8, 6, and 5 Hz) stitched together; HHT can give a better time and frequency resolution and a more accurate spectrum (Figure 1). In addition, the EMD can sift out the intrinsic undulations at different time scales, thereby preserving the time-varying properties instead of viewing the signal as a summation of infinite sinusoidal oscillations of constant amplitudes and frequencies. For physiological signals, evidence shows that IMFs can usually be associated with a specific physiologic process by matching their temporal frequency distribution to time scales at which specific mechanisms occur, such as respiratory or heart beat components in a blood pressure signal that typically are part of the overall signal (18, 19). Moreover, to prevent the potential problem caused by intermittent oscillation or perturbations frequently seen in physiological signals, a noise-assisted EMD algorithm (20) was applied to ensure that each IMF did not consist of oscillations at dramatically disparate scales. It prevented different components from overlapping in the frequency domain, which is, therefore, more appropriate for investigation of physiological signals (19). Also, an orthogonality test was performed between any pair of consecutive IMFs to further verify whether different IMFs are independent to each other.

### Usage of HHT in RR and Photoplethysmography Signals during Sleep

#### Reconstruction of Band-Specific Fluctuation

**Figure 2:**
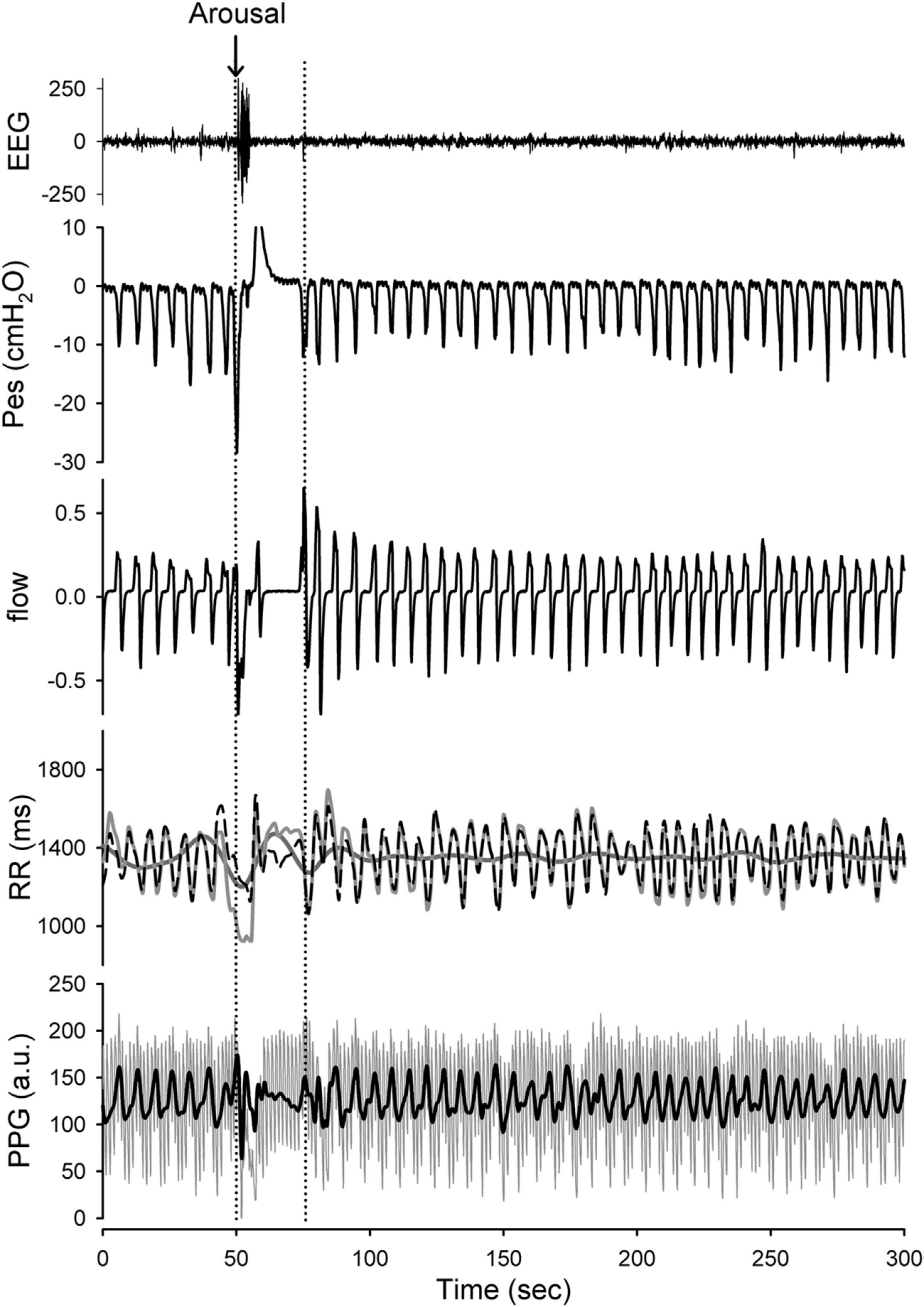
Presentation of the raw data prepared for analysis with Hilbert–Huang transform (HHT). Application of the HHT for reconstructing the instantaneous band-specific fluctuations from RR intervals and photoplethysmography (PPG) signals during stage 2 NREM sleep with increased respiratory efforts. The high-frequency (HF) component (respiratory-related fluctuation; black-dashed line) and low-frequency (LF) component (dark gray line) of the RR interval tracing (light gray line) as well as the respiratory-related component (black) of the PPG signals were determined. A transient arousal was noted in associated with the reversal of high respiratory efforts and return to stage 2 NREM sleep was recorded. The respiratory-related oscillations of the RR intervals and PPG signals can be sifted out with high temporal resolution. The respiratory-related oscillations of the RR intervals and PPG were diminished during arousal-induced central apnea and then returned to higher gain after recovery. In addition, the LF component of the RR intervals was reciprocally increased during arousal.

The R to R peaks (RR intervals) of ECG for each subject were extracted after careful visual inspection and confirmation of the validity of selection by a qualified researcher. The RR intervals and PPG signals of each patient were decomposed into different IMFs by the HHT. When decomposing the components in the RR intervals, we considered a high-frequency band (HF; 0.15–0.4 Hz) as respiratory sinus arrhythmias and modulated solely by parasympathetic control (RR_HF_), and a low frequency band (LF; 0.04 ~ 0.15 Hz) that includes activation of both sympathetic (RR_LF_) and parasympathetic controls. These findings were suggested by the studies based on the traditional spectral analysis as well as the LF/HF ratio that is thought to represent activation of sympathetic control (21). The respiratory-related IMF of RR (the temporal frequencies for its constitutive wavelets are distributed over respiratory frequency) was extracted as an instantaneous HF component and the IMFs with the temporal frequencies oscillating among the LF band were merged to represent the sympathetic and parasympathetic activations (Figure 2). In addition, we simultaneously analyzed the respiratory-related elements included in the PPG signals, and we search for the respiratory-related IMF of the PPG signal to further define and quantify the respiratory-related components (Figure 2).

#### Implementation of the Cycle-Based Analysis

**Figure 3:**
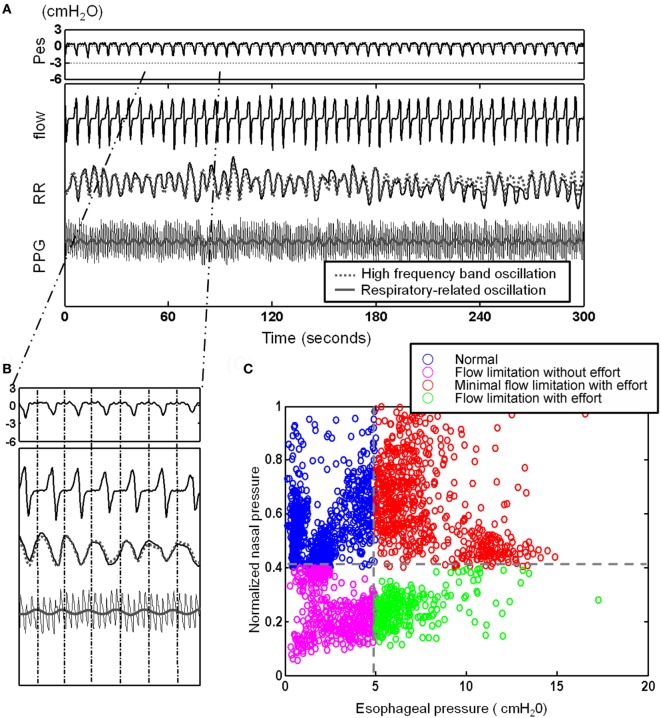
After defining the oscillations related to the respiratory-related RR oscillation [see **(A)**], here, presentation of a 5 min recording with illustration of cycle-based analysis: **(A)** 5 min segment of esophageal pressure (Pes), RR intervals (RR), photoplethysmogramy (PPG) signal, and nasal flow. Note the elicited respiratory-related oscillations of RR (gray dotted line) and PPG (gray line) by Hilbert–Huang transform. **(B)** Identification of corresponding Pes and nasal pressure cycle by cycle and quantification of cycle-based parameters: we obtained a physiologically defined cycle-based analytic tool allowing quantification of the oscillations contained within and extracted from each breath-cycle. **(C)** Four different subgroups can be identified and classified: an increase in respiratory effort indicated by more negative Pes can be associated with flow limitation or normal flow (“compensated flow” see text).

After defining the oscillations related to specific physiological controls, i.e., the respiratory-related RR oscillation (see Figure 3A), we obtained a physiologically defined cycle-based analytic tool allowing quantification of the oscillations contained within and extracted from each breath-cycle (Figure 3B).

### Cycle-Based Analysis in Assessing the Effect of Flow Limitation

**Figure 4:**
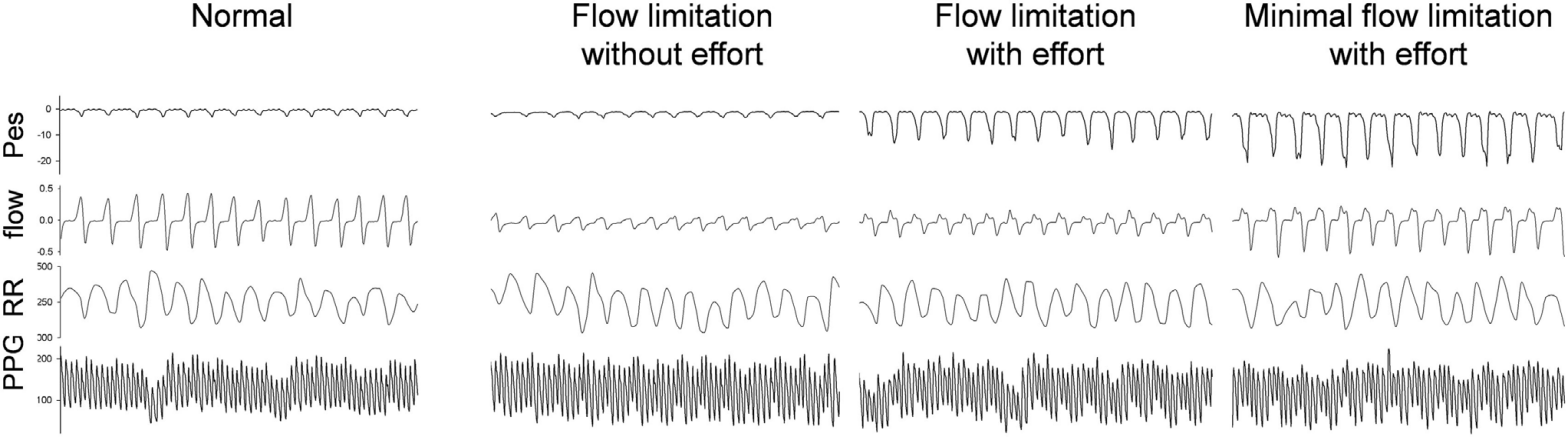
Raw data of a representative subject during stage 2-NREM sleep with different presentations of the simultaneous esophageal manometry (Pes) and the nasal flow pressure recordings. The 90-s tracings from top to bottom were Pes, nasal pressure, RR intervals, photoplethysmography (PPG) signal. The PPG signals vary in relation to the respiratory efforts as do the RR components. High respiratory efforts associated with presence of upper airway resistance syndrome independent of “compensation” to the increased effort or already presence of flow limitation at analysis of the nasal flow curve contour is associated with significant increase in parasympathetic tone.

Considering the behavior of the flow curve compared to the behavior of the Pes curve, four main patterns can be identified: Figure 4 shows the raw polysomnographic data in the four different subgroups. An increase in effort is indicated by more negative peak Pes and can be at times associated with a seemingly normal flow curve—this is interpreted as “compensation” *via* increasing inspiratory effort (i.e., the increase in effort indicated by the Pes is associated with a response in the upper airway toward maintenance of normal nasal flow). However, the progressively more negative Pes peak can also be associated with a decrease in flow (i.e., induction of “inspiratory flow limitation”). Finally, inspiratory flow limitation can also be seen without an increase in inspiratory effort, which we term “flow limitation without effort” (22).

To automatically implement the cycle-based analysis, we considered the difference between the pressures before inspiration to the most negative pressure of the Pes signal as an indicator of respiratory effort for each respiratory cycle. The flow pressure was normalized by dividing the maximal flow pressure during sleep by the flow pressure corresponding to respiratory efforts. Two thresholds were applied (−5 cmH_2_O selected as normal maximum inspiratory peak Pes and the threshold of normalized flow pressure was set at its 40 percentile) to each of the cycles and divided it as one of four different groups: normal cycles, increased respiratory efforts cycles with flow compensation (“minimal inspiratory flow limitation”), inspiratory flow limitation cycles without increased respiratory efforts [FL(−)], and inspiratory flow limitation cycles with increased respiratory efforts [FL(+)] (Figure 3C).

The computational algorithm used is as follows:

(1) The respiratory cycles, from peak-to-peak pressure of Pes signal, indicative of “maximum-inspiratory-effort” were identified. In addition, the normalized flow pressure (normalized by dividing each flow pressure by the maximal flow pressure during sleep) and the respiratory efforts (differences of the peak pressure during normal un-obstructed breathing to the most negative pressure of Pes associated with inspiratory flow limitation) were derived.
(2) We considered the SD obtained from analyses of each of the above cycles, and we derived three oscillations considered as “quantificational-parameters” for the studied physiological variables (i.e., PPG_res_ for the respiratory-related PPG oscillation, RR_HF_ for the respiratory-related RR oscillation, and RR_LF_ for the “calculated” LF RR oscillation).
(3) Each respiratory cycle was further subcategorized according to two criteria: respiratory efforts greater/less than −5 cmH_2_O and normalized flow pressure greater/less than 40 percent of the maximal flow. The respiratory cycles were then annotated into four different groups: normal cycles, increased respiratory efforts cycles with flow compensation (“minimal inspiratory flow limitation with efforts” group), FL(−), and FL(+).
(4) Finally, the averaged values of the derived parameters in the four groups were calculated for each patient.

### Statistical Analyses

The continuous variables were represented as mean values ± SD. The normality test was performed on all continuous variables. If the variables were distributed normally, Student’s paired *t-*test and repeated measures analysis of variance with Bonferroni correction were applied. Otherwise, Wilcoxon rank sum test and non-parameter repeated-measures analysis-of-variance with Tukey-HSD test were computed. A *p*-value less than 0.05 was considered statistically significant. All statistics were calculated using the open source statistical program R (version 2.15.2) (23). As a preliminary calculation, data from 15 patients not involved in the final investigation were analyzed, and sample size was calculated. The sample size of the studied parameters with a power of 0.85, alpha level of 0.05, and dropout rate of 5% were between 23 and 49 subjects. This preliminary investigation also suggested a potential subdivision of patterns in four categories “normal cycle,” FL(−), “minimal flow limitation,” and FL(+).

## Results

### Subjects

Fifty-five PSGs of subjects with well-defined clinical complaints and responding to all inclusion criteria were retrieved and 49 of these PSGs were considered valid for analyses. Six PSGs showed poor Pes signal quality during analysis of the recordings and were excluded. Nine age-matched control subjects were also retrieved. The demographic and polysomnographic data of the included subjects are summarized in Table 1. Women were predominant in both groups. UARS patients reported a presence of daytime tiredness and fatigue (100%), unrefreshing sleep (100%), difficulty concentrating (100%), light-headedness, or dizziness when abruptly standing up from a supine position particularly when getting out of bed (96%), decrease in concentration (83%), difficulty focusing (75%), memory problems (43%), and morning headaches (16%).

**Table 1:** Demographic characteristics and polysomnography results of patients with upper airway resistance syndrome (UARS) (*N* = 49) and control subjects (*N* = 9).

Variables	UARS (*N* = 49)	Normal (*N* = 9)
Age	26.8 ± 5.8	27.8 ± 4.0
Male/female	12/37	3/6
BMI, kg/m^2^	22.2 ± 2.9	24.9 ± 2.8*
Apnea–hypopnea index, no./h	3.1 ± 1.5	0.8 ± 1.1*
TST, min	411 ± 77	370 ± 39*
Sleep efficiency, %	85 ± 12	91.6 ± 6*
NREM Stage 1, %	8 ± 5	2 ± 1*
NREM Stage 2, %	61 ± 9	51 ± 6*
NREM Stage 3–4, %	16 ± 9	26 ± 5*
Stage REM	15 ± 5	19 ± 4*
HF	27.8 ± 18.2	22.5 ± 11.12*
LF	36.3 ± 16.4	32.4 ± 8.7
LF/HF	1.51 ± 0.53	1.69 ± 0.74
PPGres	5.43 ± 1.47	5.50 ± 1.80
% Flow limitation/TST	68.6 ± 11.3	5 ± 5*

*p<0.05 compared to UARS group.

*BMI, body mass index; TST, total sleep time; HF, high-frequency power; LF, low-frequency power; PPGres, respiratory-related PPG oscillation*.

Snoring was present for variable amounts of time during the recording in all subjects with UARS. All of the subjects had AHIs lower than 5 events/h but the AHIs of UARS patients were significantly higher than control subjects. Although total sleep time was significantly shorter in control subjects, the sleep efficiency and percentage of sleep time spent in stage 3 NREM sleep were markedly decreased in patients with UARS compared to control subjects (Table 1). In UARS patients, the percentage of stage 2 NREM sleep was significantly increased compared to the control group (61 ± 9 vs 51 ± 6% of total sleep time). All UARS patients had large amounts (>65% of total sleep time) of “inspiratory flow limitation” at visual scoring of PSG.

### Pes-Related Changes in Parameters Derived from Cycle-Based Analysis

Pes was significantly more negative and normalized flow was markedly lower during supine compared to non-supine positions in patients with UARS (Table 2). However, none of the derived parameters differed as a result of different sleep positions. In addition, no significant change was found between the first third and the last third of the night. There was no indication of signal drifting during total sleep time.

**Table 2:** Parameters derived from cycle-based analysis during different sleep cycles or positions.

	Position		Duration of night	
Parameters	Non-supine^a^	Supine	First third of sleep	Last third of sleep
Nasal_norm_	0.54 ± 0.16	0.45 ± 0.16*	0.51 ± 0.18	0.52 ± 0.19
Pes (cmH_2_O)	6.94 ± 2.62	7.96 ± 3.63*	7.56 ± 3.51	7.63 ± 3.71
Breath length	4.20 ± 0.54	4.18 ± 0.56	4.22 ± 0.54	4.22 ± 0.57
RR_HF_	34.7 ± 26.6	34.7 ± 24.6	39 ± 24.2	36 ± 25.8
RR_LF_	40.8 ± 19.5	40.2 ± 19.7	44.3 ± 21	39.6 ± 16
LF/HF	1.87 ± 0.75	1.72 ± 0.82	1.7 ± 1.01	1.67 ± 0.69
PPG_res_	4.14 ± 1.18	4.39 ± 1.41	4.6 ± 2.3	4.4 ± 1.3

^a^Non-supine:lateral sleeping or prone position, *p < 0.05.

*Pes, esophageal pressure; RR_HF_, respiratory-related RR oscillation; RR_LF_, “calculated” LF RR oscillation; PPG_res_, respiratory-related PPG oscillation*.

Compared to control subjects, higher parasympathetic activation of the UARS patients during stage 2 NREM sleep was noted by the RR_HF_ derived from the respiratory cycles (27.8 ± 18.2 vs 22.5 ± 11.12, *p* < 0.05). A detailed analysis indicated different findings depending on the above subdivisions in UARS group (Table 3).

**Table 3:** Parameters derived from cycle-based analysis in different Pes and flow pressure groups during NREM stage 2.

Parameters	Normal cycles group	FL(−)	Minimal flow limitation group	FL(+)
RR_HF_	33.0 ± 23.5	33.0 ± 24.7	36.2 ± 26.7*^†^	35.7 ± 23.5*^†^
RR_LF_	40.1 ± 18.3	39.9 ± 18.0	44.7 ± 25.7	39.0 ± 17.5
LF/HF	1.93 ± 0.97	2.01 ± 1.01	1.90 ± 0.91	1.66 ± 0.80*^†^
PPG_res_	4.10 ± 1.66	4.18 ± 1.55	4.46 ± 1.51*^†^	4.64 ± 1.62*^†^

*p < 0.05 compared to normal cycles group.

*^†^p < 0.05 compared to flow limitation without efforts*.

*Pes, esophageal pressure; RR_HF_, respiratory-related RR oscillation; RR_LF_, “calculated” LF RR oscillation; PPG_res_, respiratory-related PPG oscillation; FL(−), flow limitation without effort group; FL(+), flow limitation with efforts group*.

The normalized mean flow pressure was significantly lower in subgroups with “inspiratory flow limitation” cycles [0.64 ± 0.07 for normal cycle group, 0.65 ± 0.07 for minimal flow limitation group, 0.28 ± 0.06 for FL(−), and 0.27 ± 0.06 FL(+)], while the oxygen saturation extracted for each studied respiratory cycle from the finger oximetry curve (Massimo oximeter™) showed no significant difference among all groups [96.8 ± 1.1% for normal cycle group, 96.8 ± 1.1% for compensation group, 96.9 ± 1.1% for FL(−), and 96.8 ± 1.1% FL(+)]. For many of the studied variables, there was no difference between the normal cycle group and the FL(−) group.

When looking at indicators of ANS responses, different responses were noted depending on effort. During “high” (increased) respiratory efforts [i.e., minimal inspiratory flow limitation and FL(+) pattern groups], the RR_HF_ and PPG_res_ were significantly increased compared to the normal cycle and the FL(−) pattern groups. More importantly, the RR_LF_/RR_HF_ considered as an index of sympathetic activity was significantly lower in the FL(+) group than in the normal cycle and FL(−) groups (Table 3).

## Discussion

The main finding of this study is that during stage 2 NREM sleep, patients with UARS had higher parasympathetic activity when respiratory efforts were high and inspiratory flow limitation was presented as shown by instantaneous cycle-based analysis of heart rate variability derived from HHT. The augmented respiratory-related PPG fluctuations triggered by high respiratory efforts serve as an indicator of parasympathetic activation and/or withdrawal of sympathetic activation.

Disturbances of sleep induced by inspiratory flow limitation can occur without significant oxygen saturation drops, and in the absence of easier to visually scored long EEG-arousals. But the short lasting EEG changes are sleep disturbances, including an increase of the phase A2 of the cyclic alternating patterns (CAP) (5); and such increase in the phase A2 of the CAP-scoring system demonstrate brain disturbances and arousals better than the AASM scoring system that request a minimum of 3 s to score a disturbance. Inspiratory flow limitation is not well integrated into the PSG scoring of abnormal breathing during sleep because it is more difficult to score visually than a complete apnea or to score an oxygen-desaturation-index. Such scoring issues have been a handicap in the recognition of “flow limitation,” its association with short lasting EEG disturbances and ANS system impact. But a large study on a representative sample of the general population of Sao Paulo presented data on the distribution of complaining and non-complaining subjects with inspiratory flow limitation and the potential cut-off point for pathology, improving our understanding of syndromes associated with “abnormal breathing during sleep” (6). A prior limited study has also showed that compared to OSA patients, UARS patients have a higher baseline parasympathetic tone during sleep and predominantly regulate heart-rate changes during arousal (12). However, the underlying mechanisms responsible for such changes have not been reported in the literature.

The amplitude of finger PPGs decreases due to peripheral vascular constriction caused by sympathetic activation has proven to be a sensitive marker for different levels of arousal (24, 25). Heart rate and LF/HF ratio also significantly increases during arousal (12, 26). Although sleep fragmentation of the patients with inspiratory flow limitation and UARS, as evaluated by the cyclic-alternating-pattern of the NREM sleep EEG, demonstrate a higher sleep disruption in these patients compared to control subjects (5, 27), most of the flow-limitation events of those patients, as mentioned above, are not necessarily related to a visually and easily detectable arousal (5, 28).

Analysis of the changes of instantaneous heart rate (RR intervals) or pulse wave amplitude derived from PPG during those flow-limitation-events can probe the dynamic modulation of the ANS using the HHT. The presented cycle-based analysis of PPG and RR intervals quantified the signals with an adaptive basis over-time scale—cycle length of each breath. Instead of using a fixed window length, the physiologically defined window can reflect the instantaneous changes within one breath. That is, for RR intervals, the change of HF, LF, and LF/HF ratio, corresponding to every flow and esophageal pressure can be easily derived.

The activation of the parasympathetic nervous system in the cycles with high respiratory-effort, independent of changes in the flow-curve, can be explained by the impact of a more important lung stretch reflex resulting from higher negative intrathoracic pressure. Stimulation of the mechanoreceptors located in the pharyngeal area may trigger a response from controlling inspiratory neurons (29) inducing greater negative inspiratory pressure. The decreased LF/HF is only noted in inspiratory flow limitation cycles with effort but not in flow limitation cycles associated with absence or decrease in effort (monitoring of nasal cannula alone as done in many studies will not dissociate these two types of inspiratory flow limitation). Such different responses may be related to a protective mechanism triggered to maintain the patency of the upper airway involving sympathetic activation such as seen with cortical short arousal if needed, but there may be an absence of response during flow limitation with effort (i.e., presence of an incomplete adjustment that does not lead to a drop of oxygen saturation or to sufficient stimulation to trigger a “long” cortical arousal). Such absence of response may be related to different mechanisms including sleep stages, homeostatic function of sleep, and prior amount of sleep disruption (i.e., chronicity of problem, with possibly blunting of brain response if repetitive stimulation has happened for a sufficient amount of time).

Also, increased respiratory efforts can cause augmented blood pressure swings and has been shown to lead to a fall in blood pressure reading in normal and heart-transplant patients (13). Although the low frequency band of the finger PPG does not necessarily correlate to that of the arterial blood pressure, the finger PPG and the blood pressure are highly correlated to each other (30). This finding suggests that the respiratory-related control mechanisms of blood pressure can be probed by finger PPG and the above mechanisms are one of the main components of PPG fluctuation. But the amplitude of the finger PPG is sensitive to sympathetic activation (24, 25), which can mask the contributions of other mechanisms.

Independently of the above suggestions, in this study, we extracted the respiratory-related oscillations from the PPG signal to specifically focus on the possible ANS changes impacting the cardiovascular system during flow limitation and increased respiratory effort. As no significant difference in sympathetic activation can be seen in all studied groups, the augmented respiratory-related oscillation of the PPG signal can most likely be attributed to corresponding changes in blood pressure during increased respiratory effort. It is noteworthy that the relationship between the parasympathetic tone and the respiratory-related oscillation of the finger PPG may not be important in normal subjects. Nevertheless, their continuous presence in UARS subjects cannot be discarded: the clinical complaints of UARS patients support a negative long term impact of such chronic vagal stimulations with possible secondary sympathetic functional de-afferentation as demonstrated previously in experimental short-term studies manipulating breathing (13, 14). Our approach may be used to investigate patients with complex presentation such as patients with cardiac failure and central sleep apnea to better understand the BP variations in relation with breathing events.

### Study Limitation

One limitation of our study may be related to the arbitrary thresholds of Pes and nasal pressure that we set. However, the study of Pes in nine normal control subjects showed that the mean respiratory efforts according to Pes during stage 2 NREM sleep was around −4 cmH_2_O and less or equal to −5 cmH_2_O even in slow wave sleep (31). This was very similar in the nine non-complaining subjects studied here that had been monitored previously for other research protocols. In addition, the threshold for affirming inspiratory flow limitation with nasal pressure recording was not the most critical factor based on the results. We cannot overlook the possibility that some of the inspiratory flow limitation cycles without respiratory efforts may be a normal biological variant during sleep and that the frequency of these events could represent a difference between normal and pathological conditions.

Second, any analytical approach for characterizing physiological data will have some weakness. When applying the HHT, the EMD may have difficulty in distinguishing different components in a narrowband signals. In order to preserve most of the time-varying information in IMFs, when dealing with the signal with two oscillatory frequencies too close to each other, EMD could recognize these two oscillatory waves as one component oscillating with frequency modulation. In that case, a well-described adjunct approach has to be applied [e.g., EMD with a masking signal (32, 33)] to extract the two different narrowband components. However, the respiratory-related changes in heart rate or PPG signals investigated here are rather distinct and operate in relative higher frequency. Taking into consideration the above, incorporating other component into respiratory-related oscillation in this study does not seem valid and “masking” does not seems necessary. In addition, we performed orthogonality test on any pair of consecutive IMFs to eliminate the possibility that different IMFs were attributed to same mechanism.

## Conclusion

There is a group of individuals, mostly young adults, who complain of sleep disruption and daytime symptoms, and demonstrate increased vagal tone that impacts their well-being. Their PSG recordings indicate an abnormal amount of inspiratory flow limitation and nocturnal EEG disturbances not presented in the definition of OSA syndrome. Our investigation demonstrates that in such subjects, chronic stimulation of vagal tone can occur night after night explaining some of the clinical presentations and complaints. Our study also indicates how a computer analysis of a simple signal, with usage of a new algorithm easy to integrate to automatically analyze the PPG curve (commonly derived from the monitoring of oxygen saturation using pulse-oximetry), can provide important clinical information by indicating hyperactivation of sympathetic or parasympathetic resultant tone. Such repetitive vagal hyperactivation might relate to some of the daytime complaints of this subgroup of patients and warrant for further study.

## Ethics Statement

The study was performed following the guidelines of the Stanford University IRB. This study was performed on clinical and PSG data previously acquired either on patients or on subjects involved in other research protocols with signed informed consent. The retrospective signal analyses and mathematical manipulations presented here were performed on anonymized data without any direct subject involvement. The protocol was approved by the Stanford IRB.

## Author Contributions

CG conceived the protocol, participated in analysis review of all data, and wrote the article. CL collected data, did analysis, and participated in write-up. M-TL participated in analysis.

## Conflict of Interest Statement

The authors declare that the research was conducted in the absence of any commercial or financial relationships that could be construed as a potential conflict of interest.
